# Characterization of a novel theta-type replicon of indigenous plasmid pTE15 from *Lactobacillus reuteri* N16

**DOI:** 10.1186/s12866-022-02718-4

**Published:** 2022-12-12

**Authors:** Po-Wen Chen, Chuen-Fu Lin

**Affiliations:** 1 Department of Veterinary Medicine, National Chung Hsing University, Taichung, 40249 Taiwan; 2 Department of Veterinary Medicine, College of Veterinary Medicine, National Pingtung University of Science and Technology, Pingtung, 91201 Taiwan; 3 Animal disease diagnostic center, College of Veterinary Medicine, National Pingtung University of Science and Technology, Pingtung, 91201 Taiwan

**Keywords:** pTE15, theta-type replication, *Lactobacillus reuteri*, *Lactobacillus* plasmid, Replicon

## Abstract

**Background:**

pTE15 is a ~ 15-kb narrow-host-range indigenous plasmid from *Lactobacillus reuteri* N16 that does not replicate in selected *Bacillus* spp., *Staphylococcus* spp., and other *Lactobacillus* spp.

**Methods:**

Combined deletion analysis the minireplicon essential of pTE15 with replicon-probe vector pUE80 (−) to confirmed sufficient for replication and from the ssDNA intermediate detection, plasmid amplification tested by chloramphenicol treatment, and replication origin sequence analysis to delineated the novel theta-type replication of pTE15.

**Results:**

Single-stranded intermediate of pTE15 DNA was not detected in *L. reuteri*, indicating that this plasmid does not replicate via a rolling circle mechanism. The replicon of pTE15 did not display the structural organization typical of rolling-circle plasmids, nor were they similar to known rolling-circle plasmids. We further provided evidence that this plasmid applied a new mode of theta-type replication mechanism: (1) the size of this plasmid was > 10-kb; (2) the minireplicon consisted of AT-rich (directed repeat, iteron) and DnaA sequences; (3) the minireplicon did not contain double-strand origin (DSO) and essential *rep* genes, and it also showed no single-strand origin (SSO) structure; (4) the intermediate single-stranded DNA products were not observed for pTE15 replication; (5) the minireplicon did not contain a typical essential replication protein, Rep, (6) its copy number was decreased by chloramphenicol treatment, and (7) genes in pTE15 replication region encoded truncated RepA (TRepA), RepB and RepC, which were replication-associated proteins, but they were not essential for pTE15 replication.

**Conclusions:**

Collectively, our results strongly suggested that the indigenous plasmid pTE15 of *L. reuteri* N16 belongs to a new class of theta replicons.

**Supplementary Information:**

The online version contains supplementary material available at 10.1186/s12866-022-02718-4.

## Background

Probiotics are generally defined as “live microorganisms which, when administered in adequate amounts, can confer a health benefit on the host” [1]. Presently, various probiotics, including *Lactobacillus* spp., *Bifidobacterium* spp., *Saccharomyces boulardii*, *Propionibacterium* spp., *Streptococcus* spp., *Bacillus* spp., *Enterococcus* spp., and some specific *Escherichia coli* strains, have been found to confer diverse health benefits to the host [2]. Among these potential probiotics, *L. reuteri*, which was first isolated in 1962, has been recognized as an obligatory heterofermentative and endogenous lactic acid bacterial species in the GI tract of mammals [3]. Furthermore, specific *L. reuteri* strains have been found to display various beneficial effects, including the production of antimicrobial molecules such as organic acids, ethanol, and reuterin [4]. Several *L. reuteri* strains can decrease the levels of pro-inflammatory cytokines and thus promote the function and development of regulatory T cells [5]. In addition, specific *L. reuteri* strains have been shown to reduce the incidence and severity of diarrhea, prevent colic and necrotic enterocolitis, and maintain a functional mucosal barrier in humans [5–7]. Collectively, specific *L. reuteri* strains have been used to promote growth, improve the efficiency of feed utilization, prevent diarrhea, and enhance the immune system in several animals [5, 8].

As *L. reuteri* is an obligatory, heterofermentative, and endogenous probiotic species in the GI tract of mammals, some studies have attempted to develop it or other specific *Lactobacillus* species as vaccine carriers [9–11]. Notably, a vector with a narrow host range is considered an important factor, and this type of vector is less likely to be horizontally transferred between bacterial species [12]. Moreover, it is generally accepted that the replicon origins of a vector are important regions that influence their host range, and subtle point mutations in these regions have a considerable impact on the plasmid’s host range [13, 14]. Therefore, the isolation and characterization of a set of plasmids that can replicate simultaneously in *Lactobacillus* hosts would be of great advantage for the construction of genetically altered strains [15–17].

Interestingly, in our previous study, we first reported two specific *L. reuteri* strains L1 and N16, which were isolated from the small intestines of chickens, containing a 7.0-kb plasmid (pTE80) or ~ 15-kb plasmid (pTE15), respectively. The two plasmids encode genes conferring resistance to erythromycin (Em) and have a narrow host range. Therefore, we further constructed a replicon-probe vector to identify the replication regions (RR) from the indigenous plasmids of *L. reuteri* [18] and applied this replicon-probe vector to identify the replication characteristics that was not rely exclusively on host initiation factors for the RR of the pTE82 plasmid, which is also an indigenous plasmid of the poultry strain *L. reuteri* G4 [19]. We previously reported for the first time that this plasmid contained a *pal*T-type single-strand origin (SSO) from *Lactobacillus* [19].

To date, the theta-type replication plasmid in lactobacilli were including pLP60 [20], pRC12 and pRC18 [21], pRV500 [22], pREN [23], and pLJ1 [24], especially the pRC12, pRC18, pRV500, and pREN belonged to the pUCL287 subfamily. They were class A of theta replicons that must plasmid encoded Rep protein with a related origin of replication and replicate independently of DNA pol I. Most lactic acid bacteria (LAB) theta-type replicating plasmids belong to this class [25]. Notably, there are six known classes of theta replicons [15–17, 26]. However, our data suggest that pTE15 cannot be classified into any one of the six known classes of theta replicons. To our knowledge, this is the first unclassified classes of theta replicons in indigenous plasmid of *Lactobacillus* to apply the theta replication machinery.

This study aimed to identify and characterize narrow-host-range plasmid, pTE15 of *L. reuteri* N16 to facilitate the development of *L. reuteri* as a vaccine carrier and pharmaceutical applications. Understanding plasmid maintenance and replication have significant practical implications for clinical studies and bioremediation. We found that pTE15 employed a theta-type replication mechanism, and its replication relied exclusively on host initiation factors.

## Results

### Replication region of pTE15

**Fig. 1:**
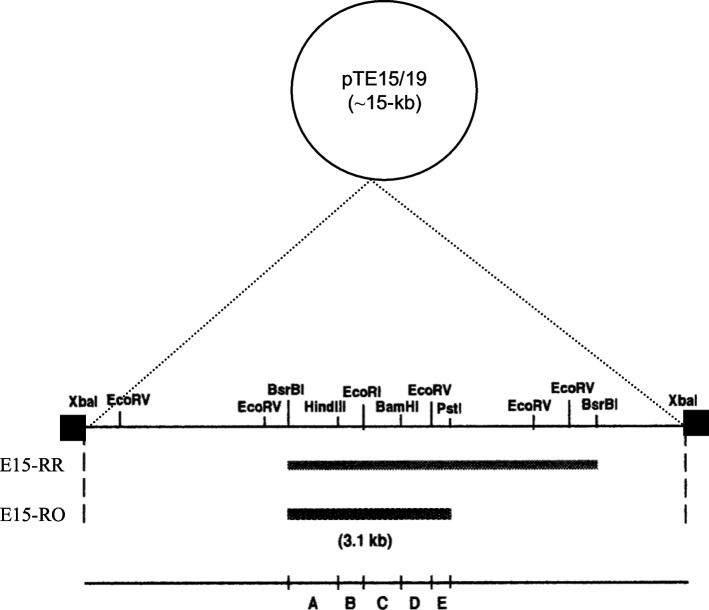
Physical map of pTE15. Cloning and subcloning of replication region of pTE15-RO for DNA sequencing

**Fig. 2:**
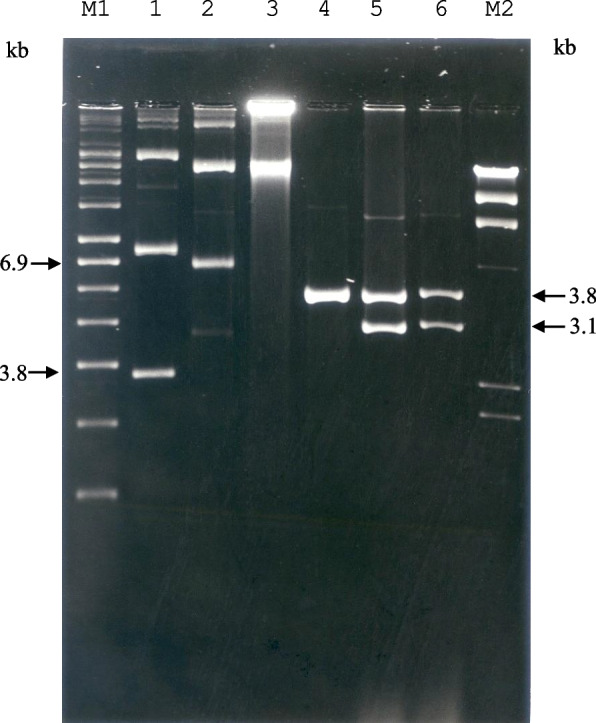
Identification of the replication region of pTE15 by DNA electrophoresis. The recombinant plasmids were undigested by restriction enzyme; lane M1: supercoiled DNA marker (Sigma-Aldrich), lanes 1, 2: pUE80 (−) (3.8-kb) and pTE15-RO (6.9-kb) were purified from *E. coli* TG1, lane 3: pTE15-RO was purified from *L. reuteri* 20016. The recombinant plasmids were digested by *Xba*I/*Pst*I, lanes 4, 5: pUE80 (−) and pTE15-RO were purified from *E. coli* TG1, lane 6: pTE15-RO was purified from recombinant *L. reuteri* DSM 20016. lane M2: λDNA/*Hin*dIII marker

To determine the essential region of pTE15 required for replication, pTE15/19 was digested with *BsrB*I, and the obtained 6.8-kb fragment was subcloned into pUE80 (−) [18], which had been digested with *Hin*cII to obtain recombinant plasmid pTE15-RR, both pTE15-RR and pUE 80 (−) were further digested with *Xba*I/*Pst*I and subcloned together to get 3.1-kb DNA fragment, named pTE15-RO. (Fig. 1 and Table 1), and they were further transformed into *L. reuteri* DSM 20016 by electroporation. The recombinant *L. reuteri* DSM 20016 can survive in a medium with Em, initially indicating that this plasmid contains the essential RR (6.8-kb and 3.1-kb fragments). Further, the identification of the RR of pTE15 was confirmed with pTE15-RO purified from recombinant *E. coli* and *L. reuteri* DSM 20016 then assessed by agarose gel electrophoresis, showing pTE15-RO contained the 3.1-kb DNA fragment could replicate in *L. reuteri* DSM 20016 (Fig. 2). Finally, the data supported that pTE15-RR and pTE15-RO contained the replicon because these derivatives helped the recombinant *L. reuteri* DSM 20016 grow well in MRS (De Man, Rogosa and Sharpe; Oxoid) medium with Em (10 μg/mL).

**Table 1:** Bacterial strains and plasmids

Strain or Plasmid	Characteristics	Source or Reference
*Lactobacillus reuteri*		
DSM 20016	Type strain, plasmid free; Cm^s^, Em^s^, Tet^s^	DSM^a^; Kandler et al. (1980) [3]
*Escherichia coli*		
TG1	Recipient in transformation; F′ *traD36*	New England Biolabs;
	*lac I*^*q*^,*Δ (lacZ)M15 proA*^*+*^*B*^*+*^*/supE*	Maniatis et al. (1989) [27]
	*Δ (hsdM-mcrB)5 (r*_*k*_^*−*^*m*_*k*_^*−*^*McrB*^*−*^*) thi*	
	*Δ (lac-proAB)*, Em^s^, Ap^s^	
Plasmids		
pUC19	*E.coli* cloning vectors; 2.7-kb, Ap^r^, *lac*Z	Yanisch-Perron et al. (1985) [28]
pTE15/19	pUC19 plus the 15-kb *Xba*I DNA	This study
	fragment from pTE15; 17.7-kb, Ap^r^, Em^r^	
pUE80 (−)	pUC19 plus the 1.0-kb *Hin*dIII-*Hin*dIII	Lin C-F. et al. (1999) [18]
	DNA fragment from pUE8010; 3.8-kb; Ap^r^, Em^r^	
pTE15-RR	pUE80 (−) plus the 6.8-kb *Bsr*BI-*Bsr*BI	This study
	DNA fragment from pTE15/19; 10.6-kb; Ap^r^, Em^r^	
pTE15-RO	Deletion derivative of pTE15RR; containing a 3.1-kb	Lin and Chung (1999) [18]
	*Bsr*BI-*Pst*I fragment of pTE15	
pTE15-RRΔ1	Deletion derivative of pTE15-RO; containing a 2.9-kb	This study
	*Bsr*BI-*Bgl*II fragment of pTE15	
pTE15-RRΔ2	Deletion derivative of pTE15-RO; containing a 2.6-kb	This study
	*Bsr*BI-*Eco*RV fragment of pTE15	
pTE15-RRΔ3	Deletion derivative of pTE15-RO; containing a 2.1-kb	This study
	*Bsr*BI-*Bam*HI fragment of pTE15	
pTE15-RRΔ4	Deletion derivative of pTE15-RO; containing a 2.3-kb	This study
	*Hind*III-*Pst*I fragment of pTE15	
pTE15-RRΔ5	Deletion derivative of pTE15-RO; containing a 2.1-kb	This study
	*Sac*I-*Pst*I fragment of pTE15	
pTE15-RRΔ6	Deletion derivative of pTE15-RO; deleting a 0.98-kb	This study
	*Sac*I-*Pvu*I fragment of pTE15-RO	
pTE15-RRΔ7	Deletion derivative of pTE15-RO; containing a 1.3-kb	This study
	*Hind*III-*Bam*HI fragment of pTE15	

^a^DSM: Deutsche Sammlung von Milroorganismen, Gottingen, FRG

Cm^s^, Em^s^, Tet^s^, Ap^s^, Ap^r^, and Em^r^ indicated the presence or not of the following antibiotic resistance genes, cholramphenicol, erythromycin, tetracycline and ampicillin

**Fig. 3:**
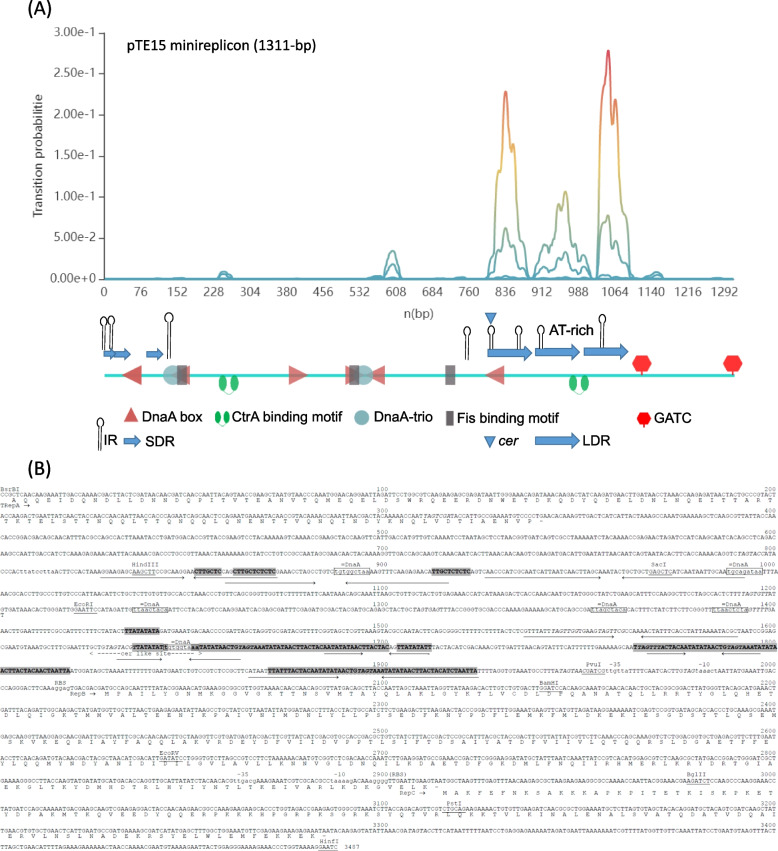
DNA sequence of the 3487-bp fragment of pTE15 containing all the information required for autonomous replication. **A** Schematic representation of the pTE15 minireplicon contained IR, SDR, LDR (AT-rich, iteron), DnaA box, and *cer-*like site. **B** The putative amino acid sequences of RepA, RepB and RepC are presented below the DNA sequence. Possible − 35, − 10, and ribosome binding sites (RBS) are presented upper the lowercase DNA sequence; inverted repeated (IR) sequences are indicated with single-line arrows; possible iteron DNA sequences (TAGTRRR) of pAD1 are presented italic word. The following characteristics are indicated: =DnaA is a DnaA-like box; cer like site is a cer-like resolution site. Regions characterized by a high AT content and including direct repeats are bolded and shaded, except the CTTGCTCTCTC sequence. The following short ORFs, lacking a potential RBS at an appropriate distance from the putative start codon, are present upstream of the transcribed repB and repC: (i) lower strand, position 1029–901, MLMGQGQGAF-KLSALQLLMS-SAAVFAKLIN-DCDGLTERAM-FS*; (ii) lower strand, position 1127–1023, MMVSHSSNSL-ICCLLNKKNQ-PAEQGLGGNT-RACE*; (iii) upper strand, position 1149–1238, MGYLKHFQGF-LATLLVLCDK-HWDWNSIDC*; (iv) upper strand, position 1281–1442, MRLRCRATAS-EFTGCDPKEK-ACSRLATHFL-SSSGFNSSFD-NLNFSPFLSI-LYI*

Next, we conducted sequence and functional analysis of the RR (3487-bp) of the *L. reuteri* plasmid pTE15, which was inferred from DNA sequence analysis (Fig. 3B). From the RR sequence of pTE15 was aligned with non-redundant sequences in NCBI, result shown the most similarity plasmid is pLRI04 and the nucleotides (1–1534-bp) at upstream of replication origin (directed repeat, iterons) of pTE15 has 95.8% identities with plasmid pLRI04 of *L. reuteri* I5007 isolated from health pig (Supplementary Fig. 1) (unpublished data).

For the DNA sequence analysis (Fig. 3B), the putative amino acid sequences of non-essential truncated RepA protein (TRepA) in the upstream of replication origin and RepB and RepC are presented below the replication origin. Possible − 35, − 10, and ribosome binding sites (RBS) are presented above the lowercase DNA sequence, which were predicted by blastx [29].

The 112 amino acid sequence of truncated RepA (TRepA) protein shared 99% identity with C-terminal residuals of Rep proteins of pRK11281 (Accession No. WP_016497314, unpublished) in *Limosilactobacillus reuteri*. RepB (295 aa) and RepC (123 aa) were also had 99% identities with AAA family ATPase (Accession No. WP_098035017) and replication-associated protein RepC (WP_098035018), respectively in *Limosilactobacillus antri* (unpublished), but these putative Rep proteins were not essential for pTE15 replication.

The replication origin had inverted repeat (IR) sequences are indicated with single-line arrows; directed repeat (DR) sequences are indicated with shadows and bold words; possible iteron DNA sequences (TAGTRRR) of pAD1 [30] are presented in italics. The following short ORFs, lacking a potential RBS at an appropriate distance from the putative start codon, are presented upstream of the transcribed repB and repC: (i) lower strand, position 1029–901, MLMGQGQGAF-KLSALQLLMS-SAAVFAKLIN-DCDGLTERAM-FS*; (ii) lower strand, position 1127–1023, MMVSHSSNSL-ICCLLNKKNQ-PAEQGLGGNT-RACE*; (iii) upper strand, position 1149–1238, MGYLKHFQGF-LATLLVLCDK-HWDWNSIDC*; and (iv) upper strand, position 1281–1442, MRLRCRATAS-EFTGCDPKEK-ACSRLATHFL-SSSGFNSSFD-NLNFSPFLSI-LYI*.

### Nucleotide sequence accession number

Replication region (3487-bp) and the full-length sequence (15322-bp) the entire plasmid pTE15 were deposited into NCBI GenBank with accession number AF036766 and submission ID: 2637896.

### Minireplicon of pTE15

**Fig. 4:**
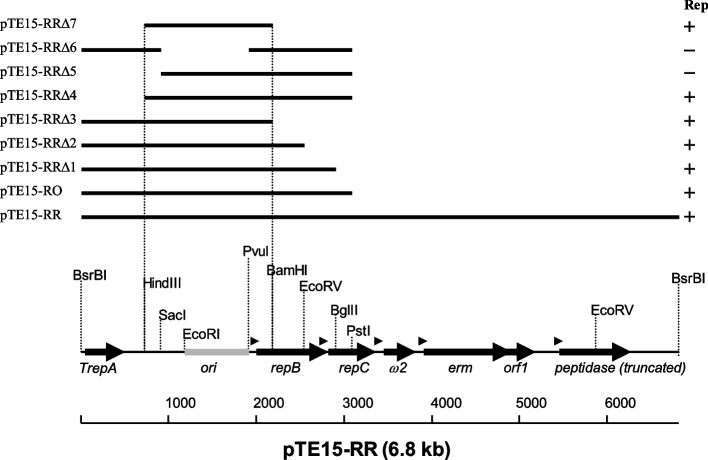
Functional analysis of the replication region of the *L. reuteri* plasmid pTE15. A linear restriction map is shown at the bottom of the diagram. Putative ORFs (black arrows), *cis*-acting region (light grey box), and promoters (arrowheads) shown on the restriction map were inferred from sequence analysis. Think lines above the linear map represent the fragments cloned into pUE80 (−). The names of recombinant plasmids containing these inserts are indicated on the left. The replication phenotype (Rep) is indicated on the right. The vertical lines delimit the core replicon

The functional analysis of the RR of the *L. reuteri* plasmid pTE15, which was inferred from the DNA sequence analysis shown in Fig. 3B. As shown in Fig. 4, putative ORFs (black arrows), *cis*-acting regions (light gray box), and promoters (arrowheads) are shown on the linear restriction map.

To further determine and confirm the minireplicon of pTE15 required for replication, we constructed a series of recombinant plasmids with different deletions, including pTE15-RRΔ1 to pTE15-RRΔ7 (Table 1), and this set of recombinant plasmids was also compared and is shown in Fig. 4. The resulting plasmids were tested for their ability to replicate and maintain in recombinant *L. reuteri* DSM 20016 with Em selection. We observed that recombinant *L. reuteri* DSM 20016 with pTE15-RRΔ5 and pTE15-RRΔ6 could not grow in a medium with Em (10 μg/mL), and the other recombinant *L. reuteri* DSM 20016 survived in a medium with Em, indicating that the other recombinant plasmids contained the RR. Collectively, compared with the deletion set of recombinant plasmid derivatives, we confirmed that pTE15-RRΔ7 contains the essential minireplicon of pTE15 required replication. Comparison of pTE15-RRΔ4 and pTE15-RRΔ5 shown the sequence between *Hin*dIII and *Sac*I cutting site, the three IR, DnaA box, and three short DR (SDR, 5′-TTGCTC-3′) (Fig. 3) were essential for pTE15 replication.

This replication origin in pTE15-RRΔ7 contained 1311-bp was genetic analysis by GCG Wisconsin Package (UWGCG) [29, 31] and DoriC 10.0 bioinformatics (http://tubic.org/doric/ and http://tubic.tju.edu.cn/doric/) [32] revealed that this region contained eight IR, three SDR and three AT-rich long DR (LDR) sequences (51-bp, interon), as well as six structures (=DnaA), three Fis binding motif, two DnaA-trio, two CtrA binding motif, and 2 GATC as shown in Fig. 3A. In addition, this region also displayed one structure (cer-like site) that was similar to the pColE1 *cer* sequence.

**Fig. 5:**
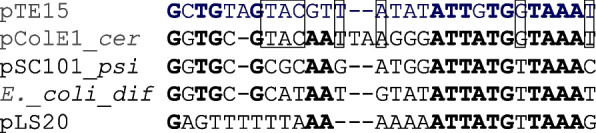
Potential site-specific recombination site of pTE15. Alignment between the RecA-independent recombination sites *dif* of the *E. coli* chromosome, *cer* and *psi* of the *E. coli* plasmids pColE1 and pSC101 respectively and the homologous region of pTE15. Identical sequences are bolded and identical sequences of pTE15 and pColE1 *cer* are boxed.

The potential site-specific recombination of pTE15 was also compared with that of the *E. coli* chromosome and plasmid, as shown in Fig. 5. A few identical sequences were recognized between the *cer*-like site of pTE15 (position 1624–1652 in Fig. 3B, 5’-GCTGTAGTACGTTATATATTGTGGTAAAT-3′) and the chromosome of *E. coli* (RecA-independent recombination site, *dif*), pColE1 (*cer*), and pSC101(*psi*) of *E. coli*. The highly identity of pTE15 *cer*-like site and pColE1 *cer* was 71.4% with gap-excluded, but a short DNA sequence of 12-bp within the minoreplicon (position 1641–1652) was 91.7% identity of them (Fig. 5).

Collectively, the above data support that pTE15 employ a theta form replication mechanism that could be a class B (pColE1 type) mode that was not necessary plasmid encoded Rep protein for ColE1 replication. Thus, we performed further experiments to dissect these issues, as explained below.

### Absence of single-stranded DNA in cells harboring pTE15

**Fig. 6:**
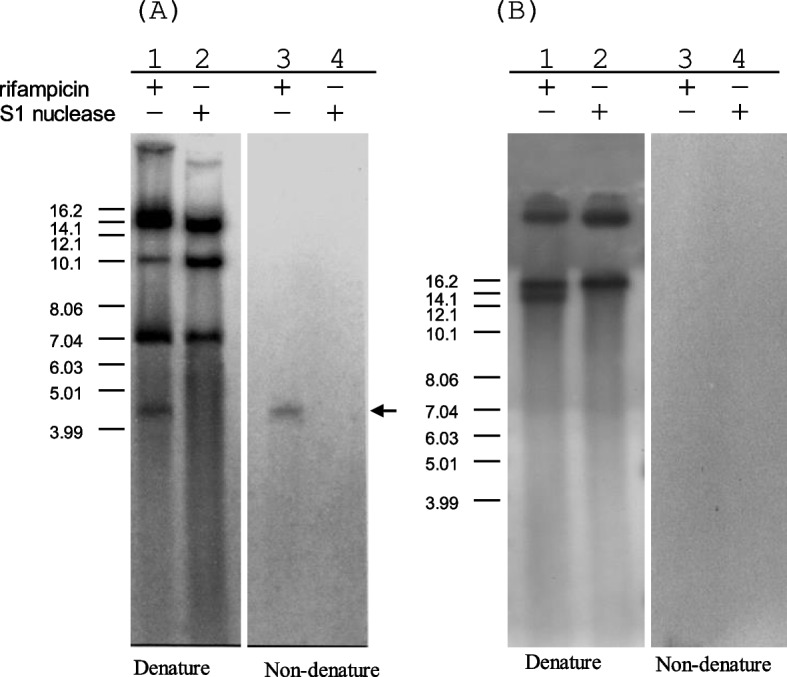
Detection of ssDNA from pTC82-RO (**A**) and pTE15 (**B**) by Southern hybrisization. Total DNA from pTC82-RO or pTE15, grown with (+) or without (−) rifampicin, was electrophoresed on a 0.8% agarose gel with (+) or without (−) prior S1 nuclease treatment. Arrow indicated the position of ssDNA intermediates. Size standards of supercoiled DNA are indicated on the left

Rolling-circle replication (RCR) plasmids are known to produce ssDNA replication intermediates [33, 34]. Thus, to determine whether pTE15 generates ssDNA intermediates during plasmid replication, we conducted a Southern blot analysis of pTC82-RO and pTE15 plasmids. Recombinant pTC82-RO was selected as a positive ssDNA control based on our previous study [19]. As expected, ssDNA was detected in the culture of pTC82-RO grown with rifampicin and was degraded when S1 nuclease was added to the cell lysates (Fig. 6A). In contrast, for pTE15, no single-stranded pTE15 DNA was detected, even after prolonged exposure to the film (Fig. 6B). Thus, the absence of single-stranded intermediate DNA of pTE15 indicated that this plasmid did not replicate via the RCR mechanism.

### Dissection for class B form of theta replication

The data in Figs. 3 and 4 imply that pTE15 may employ a class B theta form of replication mechanism. The class B form of theta replication is known to be dependent on DNA polymerase I, and plasmid DNA yields can be elevated by adding chloramphenicol (Cm) to the culture medium to amplify plasmid copy numbers (PCN) [35, 36]. Therefore, we further determined variations in the PCN of pTE15 amplification with the addition of Cm to the culture (Table 2 and Supplementary Fig. 2). pUC19 as the positive control was transformed into *E. coli* TG1 and pTE15 was transformed into *L. reuteri* DSM 20016, when they grow in the mid-log phase, and then cultured with Cm (150 μg/mL). The plasmid amplification statuses of pUC19 and pTE15 were absolute quantification by qPCR. As expected, the PCN of pUC19 was considerably mild increased after the addition of Cm to the culture (Table 2). In contrast, the amplification efficiency of pTE15 was decreased by the addition of Cm to the culture, the PCN of pTE15 from 5.96 decreased to 2.35 after Cm treatment (Table 2).

**Table 2:** Estimated plasmid copy number (PCN) by absolute quantification

Culture	C_T_^a^		Copies^b^ (copies/μL)		PCN^c^ pUC19
	*bla*	*dxs*	*bla*	*dxs*	
Before Cm^d^	7.96 ± 0.02	16.23 ± 0.03	1.78 × 10^8^ (2.5%)	6.70 × 10^5^ (1.8%)	245.1 (4.2%)
After Cm	7.34 ± 0.04	16.00 ± 0.09	2.70 × 10^8^ (5.4%)	8.15 × 10^5^ (5.6%)	325.6 (1.7%)
	*repB*	*alr*	*repB*	*alr*	pTE15
Before Cm^d^	9.63 ± 0.04	12.33 ± 0.07	5.80 × 10^7^ (4.1%)	9.50 × 10^6^ (5.6%)	6.0 (1.7%)
After Cm	12.27 ± 0.06	13.49 ± 0.03	9.90 × 10^6^ (4.8%)	4.36 × 10^6^ (2.2%)	2.3 (4.3%)

^a^Average ± S.D. (*n* = 3)

^b^Average (coefficient of variation)

^c^Average ± S.D. (*n* = 3)

Collectively, we demonstrated that the minireplicon of pTE15 contains the characteristics of IR, SDR, AT-rich (LDR, interon), DnaA box, and ColE1 *cer*-like sequences; however, this plasmid cannot be further amplified by adding Cm.

### Stability of plasmids containing the cloned RR

**Fig. 7:**
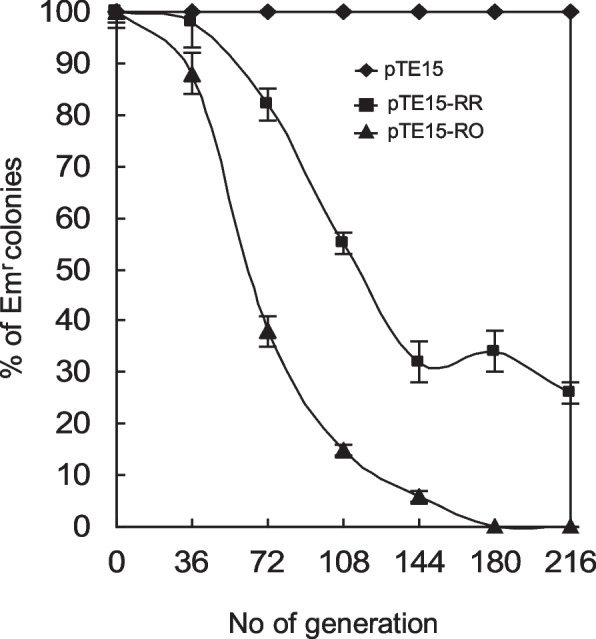
Detection of replication stability of pTE15, pTE15-RR, and pTE15-RO. No. of generations are indicated without antimicrobials selective pressure. Percentage (%) of Em^r^ colonies are expressed as the ratio (%) of erythromycin-resistant CFU scored after growth for the indicated number of generations

To evaluate the stability of the cloned RR and original plasmid pTE15 (~ 15-kb), pTE15-RR (6.8-kb), and pTE15-RO (3.1-kb) were subjected to stability analysis (Fig. 7). After 216 generations of growth without antimicrobial selective pressure, 100% of Em-resistant colonies were observed for recombinant *L. reuteri* DSM 20016 containing pTE15. However, approximately 30% of these colonies were detected in recombinant *L. reuteri* containing pTE15-RR. In contrast, only 5% were observed for recombinant *L. reuteri* DSM 20016 containing pTE15-RO after growing for approximately 144 generations (Fig. 7).

## Discussion

Most small and multi-copy plasmids of gram-positive bacteria are known to replicate via an RCR mechanism, and these RCR plasmids replicate with ssDNA intermediates. In contrast, most gram-negative bacteria replicate via the theta mechanism. In the present study, pTE15 was found to be a ~ 15-kb large plasmid from *L. reuteri* N16, suggesting that it may replicate via the theta mode. This study describes the characterization of pTE15 and showed that this large, naturally occurring plasmid (~ 15-kb) replicates via another theta mode mechanism. To our knowledge, although the characteristics of six theta-type plasmids belonged to lactobacilli were description thus for [20–24], but they almost could be classified as class A replicon, which must the plasmid encoded the Rep protein with a related origin of replication and replicate independently of DNA polymerase I [25]. Therefore, this is the first study demonstrating an unclassified class replicon of theta plasmid in a *Lactobacillus* species. The evidence for theta replication of pTE15 was based on the following observations: (1) unlike RCR plasmids, pTE15 did not generate any ssDNA replication intermediates; (2) the size of this plasmid was > 10 kb; (3) the *ori* consists of 8 IR, 3 SDR, an AT-rich (3 LDR) and 6 DnaA sequence; (4) it did not contain any features consistent with RCR. For example, it did not contain the DSO and *rep* genes and showed no SSO structure; (5) the essential replication motif of pTE15 does not contain a typical replication protein, Rep, and (6) its PCN was decreased by Cm treatment.

Six classes of theta-type plasmids can be distinguished based on their mode of replication initiation, including class A to F [15–17, 26]. Class A theta plasmids include R1, RK2, R6K, pSC101, pPS10, F, and P. These plasmids depend on plasmid encoded Rep proteins for replication initiation [37]. Class B theta plasmids include pColE1 and pColE1-like plasmids, and these plasmids can be amplified by adding Cm to the medium during the mid-log growth phase of bacteria. Most importantly, these plasmids rely exclusively on host factors for both double-strand melting and primer synthesis; thus, their replication does not depend on plasmid-encoding Rep proteins [38]. Class C plasmids include pColE2 and pColE3, and their replication depends on the Rep protein. Moreover, the Rep protein in class C plasmids displays primase activity, synthesizing a unique primer RNA (ppApGpA) that is extended by DNA polymerase I at a fixed site in the origin region [39]. Class D includes large, low-copy streptococcal plasmids that replicate in a broad range of gram-positive bacteria, including pAMβ1 from *Enterococcus faecalis*, pIP501 from *Streptococcus agalactiae*, and pSM19035 from *Streptococcus pyogenes* [40]. Replication of these plasmids also requires the plasmid-encoding Rep protein and DNA polymerase I [41]. Class E was represented by plasmid pLS20 from *Bacillus subtilis* that are not yet to be characterized [42]. Finally, class F must a replication initiation protein (RepN) but lack AT-rich region at replication initiation site and had multiple interons (DR) on the coding sequence of RepN. Its replication is independent of DNA polymerase I, such as pLJ1 of *Lactobacillus* sp., pAD1, pCF10, pPD1 and so on [25].

Based on the above classifications and characteristics of the six classes of theta replication, pTE15 could not be classified into either of them. Thus, we suggest that pTE15 be classified into a new theta mode of replication. The characteristics of this new theta replicon of pTE15 are discussed below.

According to our genetic analysis of pTE15 *ori*, the replicon of pTE15 contained unique features. For example, the iterons region with AT-rich sequences should be the *ori*; however, there were quite a few long sequences of IR around these AT-rich regions (Fig. 3A). These features have not been reported in current publications. For example, most of the motifs in the AT-rich region are DR of most prokaryotic plasmids, although there are exceptions such as RK2 the *ori*V origin, or *Thermotoga maritima*, where one of the motifs is inverted in relation to the others [43]. Another feature of pTE15 is that the essential *ori* is only about 1.3-kb (pTE15-RRΔ7, Table 1 and Fig. 4), which is not long enough to be translated for 60 amino acid residues (Fig. 3B). To support this, genetic analyses demonstrated that there was no ORF in this region. Moreover, pTE15 did not only generate any ssDNA replication intermediates but also this PCN could be decreased by adding Cm to the culture medium after mid-log phase. Thus, pTE15 cannot be classified under the class B mode of theta replication. However, according to our genetic analysis of pTE15 *ori*, it contains 6 DnaA box and an AT-rich (iterons, LDR) sequence that resembles *an ori*A-like structure. Collectively, the unique features of the minireplicon of pTE15 imply that this plasmid is a narrow-host-range plasmid. To support this, we showed that pTE15 was found in recombinant *L. reuteri* DSM 20016 at a copy number of approximately 6.0 per chromosome equivalent by qPCR. Furthermore, pTE15-RO functions only in *L. reuteri* DSM 20016 and *L. fermentum* PO2 bacterial strains, but does not function in several bacterial strains, including *L. acidophilus* ATCC 43258, *L. plantarum* ATCC 14917, *L. lactis* ATCC19435, *B. subtilis* RM125, *B. circulans* ATCC 21738, *S. epidermidis* BO95, *S. aureus* CNCTC 99/85, and *E. faecalis* JH2–2 [18]. Thus, pTE15 features as a low-copy number and narrow-host-range plasmid.

Altogether, our present data strongly suggest that pTE15 can be classified into the theta mode of replication, but it does not match the six previously known classes of theta mode replication mechanisms. Interestingly, the class E pLS20 plasmid from the gram-positive bacterium *B. subtilis* is a prototype conjugative plasmid, and its replication is shown to be fundamentally different from that of other plasmids in that it is unusually small (∼1.1-kb) and does not contain a typical replication gene [44]. Moreover, pLS20-derived replicons were established in a Pol I-mutated strain, and its copy number was not affected by Cm treatment. Therefore, the replication of pTE15 here is similar to that of pLS20, but pTE15 also contains unique features that have not been observed in the present study, as explained elsewhere. However, there is currently no DNA polymerase I mutant strain for *L. reuteri*, and thus, whether the host DNA polymerase I is involved in it replication initiation remains unclear.

Genetic analysis of pTE15 showed that the upstream of *ori* encoded 112 amino acid sequence of truncated RepA protein (TRepA) shared 99% identity with C-terminal residuals of Rep proteins of pRK11281 (Accession No. WP_016497314, unpublished) in *Limosilactobacillus reuteri*. The downstream of *ori* contained RepB (295 aa) and RepC (123 aa) were also had 99% identities with AAA family ATPase (Accession No. WP_098035017) and replication-associated protein RepC (WP_098035018), respectively in *Limosilactobacillus antri* (unpublished). Although TRepA, RepB and RepC were associated with pTE15 replication but these putative proteins were not essential for pTE15 replication. Otherwise when compared RepB and RepC of pTE15 with RepB and RepC of pAD1 using Blastp [29], which previous had been functional study [29], the similarity for amino acids was approximately 57 and 32%, respectively. The RepB involved in plasmid copy control and RepC may be involved in stable inheritance [29]. Thus, the functions of RepB and RepC in pTE15 could be similar to those of RepB and RepC in pAD1. Our stability analysis indicated that pTE15 is quite stable because it grew for 216 generations with 100% stability, without antimicrobial selective pressure. In contrast, only 5% of Em-resistant colonies were observed for *L. reuteri* DSM 20016 containing pTE15-RO after growing for approximately 144 generations. Since pTE15-RO lacks the complete *rep*C sequence, these results imply that the *rep*C of pTE15 is also involved in the stability of pTE15.

## Conclusions

In conclusion, we described the features of an indigenous pTE15 plasmid from *L. reuteri* N16, and our data suggest that this plasmid should be classified as a distinct theta-type class in lactobacilli.

## Methods

### Bacteria and growth conditions

*All the bacterial strains, indigenous plasmids, and recombinant plasmids used in the study are listed in* Table 1*. Briefly, Lactobacillus reuteri* DSM 20016, a type of strain that contains no plasmid and also possesses Cm sensitive (Cm^s^), Em sensitive (Em^s^), and tetracycline sensitive (Tet^s^) abilities was selected and grown according to a previous study [3]. *Escherichia coli* TG1 (New England Biolabs) was cultured in LB broth (Luria-Bertani; Gibco) at 37 °C. When required, ampicillin (100 μg/mL; Sigma) and Em (10 μg/mL for *Lactobacillus* and 150 μg/mL for *E. coli*; Sigma) were added to the culture media. Notably, *L. reuteri* DSM 20016 transformants were selected for growth on MRS (De Man, Rogosa and Sharpe; Gibco) with Em (10 μg/mL) to contain a cloned fragment on which the replication function was located. Stocks of bacterial strains were maintained in 15% glycerol at − 80 °C. Other information regarding the recombinant plasmids is described in detail in Table 1.

### Plasmid extraction and electrophoresis

Molecular manipulations, such as genome and plasmid isolation, electrophoresis, restriction endonuclease digestion, and fragment ligation, were performed according to standard techniques [27]. Large-scale plasmid DNA was purified using two successive cesium chloride (CsCl) density gradients [27]. Gel electrophoresis was performed on 0.8% agarose gel in TAE buffer (40 mM Tris-acetate, 1 mM EDTA; pH 7.0) at 5 V/cm.

### Plasmid constructions and confirmation of the existence of minireplicon

The characteristics of the plasmids used, including pUC19, pUE80 (−), and pTE15, pTE15-RO, were generated as described in our previous study (Lin and Chung 1999). Briefly, to generate pTE15/19, pTE15 (~ 15.0 kb) was digested with *Xba*I, and pUC19 was digested with *Xba*I and calf-intestinal alkaline phosphatase. The two plasmids were cloned to obtain pTE15/19 (Fig. 1). Next, according to the mapping of pTE15 (Fig. 1), pTE15/19 was further digested with *Bsr*BI, and the obtained 6.8-kb fragment was subcloned into pUE80 (−) that had been digested with *Hinc*II. The resulting recombinant plasmid was named pTE15-RR. pTE15-RR was then electroporated into *L. reuteri* DSM 20016 and screened using Em (10 μg/mL). Because the recombinant *L. reuteri* DSM 20016 with pTE15-RR could survive treatment with Em, it was digested with pTE15-RR and *Xba*I/*Pst*I to generate a 3.1-kb fragment. This fragment was further subcloned to generate pTE15-RO, which contains the potential replication region (RR), and was subcloned into five fragments (A–E) for DNA sequencing (Fig. 1) [18]. To further confirm which fragments may contain the needed regions of replication origins, pTE15-RO was digested and subcloned as described below.

To examine and characterize its minireplicon, a deletion approach was employed in the present study. Briefly, pTE15-RO was further digested using different restriction enzymes to delete one of its seven fragments, generating pTE15-RRΔ1 to pTE15-RRΔ7 (Fig. 4). For example, the pTE15-RRΔ1to RRΔ7 regions were deleted using *Bsr*BI-*Bgl*II, *Bsr*BI-*Eco*RV, *Bsr*BI-*Bam*HI, *Hin*dIII-*Pst*I, *Sac*I-*Pst*I*, Sac*I-*Pvu*I*,* and *Hind*III-*Bam*HI, respectively (Table 1). Then, pTE15-RRΔ1 with different deleted regions was further subcloned into pUE80 (−) and then transformed into *E. coli* TG1 to confirm the correctness of these fragments. Then, these recombinant plasmids were purified and electroporated into *L. reuteri* DSM 20016 and screened using Em (10 μg/mL) to confirm which pTE15-RRΔ1 - RRΔ7 was responsible for replication. For instance, if recombinant *L. reuteri* DSM 20016 with transformed pTE15-RRΔ1 can grow under Em treatment, then pTE15-RRΔ1 contains the machinery of the RR. The integrity of the modified fragments in each constructed plasmid derivative was confirmed using DNA sequence analysis.

### Analysis of ssDNA intermediate production

Single strand DNA was detected in the total DNA of *L. reuteri* transformed with pTE15 according to our previous study [19].

### DNA sequencing

Universal primer was used according to the procedures described in the Sequenase 2.0 enzyme kit (US Biochem). Analysis of DNA sequence with gene finding and pattern recognition was performed using Blastx and GCG Wisconsin Package (UWGCG) provided by the Genetics Computer Group [29, 31].

### Determination of absolute plasmid copy number

The plasmid copies number (PCN) of pUC19 and pTE15 were measured using quantitative real-time PCR (qPCR). Plasmid pUC19 was extracted from *E. coli* TG1 using the QIAGEN Plasmid Mini kit (Qiagen) and total DNA was extracted from *E. coli* contained pUC19 and *L. reuteri* DSM 20016 contained pTE15 using the QIAamp DNA Mini kit (Qiagen) and the method reported by Alimolaei and Golchin [45], respectively. The high purity of pUC19 was quantified by NanoPhotometer N60 (IMPLEN), then diluted continuously and the 10-fold dilution (10^1^–10^7^ copies/μL) was utilized to construct standard curves of *bla* for absolute quantification (Supplementary Fig. 2). Amplification and detection single copy genes were *bla*, *dxs*, *rep*B, and *alr* of pUC19, *E. coli*, pTE15, and *L. reuteri*, respectively. The PCN of pUC19 and pTE15 were then calculated by dividing the copy number of *bla* and *repB* by the copy number of *dxs* and *alr*, respectively. All qPCRs were conducted three times, and the average revalues were used to calculate PCN.

### Measurement of plasmid stability

A stability study was conducted according to a previous study with some modifications (Weaver, Clewell et al. 1993). Briefly, recombinant *L. reuteri* DSM 200016 containing pTE15, pTE15-RR, or pTE15-RO was grown overnight in MRS medium containing Em (10 μg/mL), which was further diluted 10^2^-fold in antimicrobial-free MRS broth (time zero) and maintained in log phase from that point by periodic dilution every 10 generations (ca. 7.5 h). At each 36-generation interval (ca. 27 h), portions of the bacterial culture were analyzed for the percentage (%) of colonies that still maintained the plasmid-encoded Em resistance. All plasmid stability tests were conducted three times.

## Supplementary Information

**Additional file 1: Supplementary Figure 1.** Alignment of pTE15 replication region (RR) with non-redundant sequences in NCBI was blast tree by Tree Viewer 1.19.3. The blast tree shown the most similarity plasmid is pLRI04 and the nucleotides (1–1534-bp) at the upstream of iteron (LDR, AT-rich) of pTE15 has 95.8% identities with plasmid pLRI04 of *L. reuteri* I5007 isolated from health pig (unpublished data). **Supplementary Figure 2.** Construction of the standard curve for *bla* and confirmation of qPCR amplification specificities of *bla*, *dxs*, *repB*, and *alr*. (A) The standard curves were constructed with seial 10-fold dilutions of the pUC19, ranged from 1 × 10^1^ to 1 × 10^7^ copies/uL. Each standard dilution was amplified by real-time qPCR using *bla*-set in eight duplication. For each gene, determined C_T_ values were plotted against the logarithm of their known initial copy numbers *(n* = 3). A standard curve was generated by linear regression through these points. (B - E) Melting peaks were examined for the *bla*-, *dxs*-, *repB*-, and the *alr*-sets with a quantitative standard sample, *E. coli* and *L. reuteri* total DNA samples before and after Cm treatments as templates. The melting temperatures were in the panels B to E. Each DNA sample was amplified by real-time qPCR in triplicate.

**Additional file 2: Figure S6.** (A) of original blots were from the red box with dash-line in lines 3–4 and lines 7–8 of panel C. (B) of original blots were from the blue box with dash-line in lines 1–2 and lines 3–4 of panel D. In panel D, lines 5–8 were from RCR-type plasmid pC194 of *Staphylococcus aureus*, which was not included in this manuscript.

## Data Availability

the data (nucleotide sequence) is deposited on NCBI GenBank (Accession No. OP744582 and AF036766).

## Abbreviations

Em: Erythromycin
Cm: Chloramphenicol
Tet: Tetracyclin
DSO: Double-strand origin
SSO: Single-strand origin
RCR: Rolling-circle replication
RBS: Ribosome binding sites
SDR: Short directed repeat
LDR: Long directed repeat
IR: Inverted repeat
RR: Replication region
LAB: Lactic acid bacteria
PCN: Plasmid copy number
