# Supplementary material for: Characterization of a novel theta-type replicon of indigenous plasmid pTE15 from Lactobacillus reuteri N16
Source: BMC Microbiol. 2022 Dec 12;22:298. doi: 10.1186/s12866-022-02718-4 (PMC9743546; doi:10.1186/s12866-022-02718-4)
Supplement: Supplementary file 1 — Additional file 1: Supplementary Figure 1. Alignment of pTE15 replication region (RR) with non-redundant sequences in NCBI was blast tree by Tree Viewer 1.19.3. The blast tree shown the most similarity plasmid is pLRI04 and the nucleotides (1–1534-bp) at the upstream of iteron (LDR, AT-rich) of pTE15 has 95.8% identities with plasmid pLRI04 of L. reuteri I5007 isolated from health pig (unpublished data). Supplementary Figure 2. Construction of the standard curve for bla and confirmation of qPCR amplification specificities of bla, dxs, repB, and alr. (A) The standard curves were constructed with seial 10-fold dilutions of the pUC19, ranged from 1 × 101 to 1 × 10^7^ copies/uL. Each standard dilution was amplified by real-time qPCR using bla-set in eight duplication. For each gene, determined CT values were plotted against the logarithm of their known initial copy numbers (n = 3). A standard curve was generated by linear regression through these points. (B - E) Melting peaks were examined for the bla-, dxs-, repB-, and the alr-sets with a quantitative standard sample, E. coli and L. reuteri total DNA samples before and after Cm treatments as templates. The melting temperatures were in the panels B to E. Each DNA sample was amplified by real-time qPCR in triplicate. [file 12866_2022_2718_MOESM1_ESM.docx]

Supplementary Figure 1.

Lactobacillus reuteri N16 plasmid pTE15, replication region

0.05

Supplementary Figure 2.

**Supplementary Figure 1.** Alignment of pTE15 replication region (RR) with non-redundant sequences in NCBI was blast tree by Tree Viewer 1.19.3. The blast tree shown the most similarity plasmid is pLRI04 and the nucleotides (1-1534-bp) at the upstream of iteron (LDR, AT-rich) of pTE15 has 95.8% identities with plasmid pLRI04 of *L. reuteri* I5007 isolated from health pig (unpublished data).

**Supplementary Figure 2.** Construction of the standard curve for *bla* and confirmation of qPCR amplification specificities of *bla*, *dxs*, *repB*, and *alr*. (A) The standard curves were constructed with seial 10-fold dilutions of the pUC19, ranged from 1 × 10^1^ to 1 × 10^7^ copies/uL. Each standard dilution was amplified by real-time qPCR using *bla*-set in eight duplication. For each gene, determined C_T_ values were plotted against the logarithm of their known initial copy numbers *(*n=3). A standard curve was generated by linear regression through these points. (B - E) Melting peaks were examined for the *bla*-, *dxs*-, *repB*-, and the *alr*-sets with a quantitative standard sample, *E. coli* and *L. reuteri* total DNA samples before and after Cm treatments as templates. The melting temperatures were in the panels B to E. Each DNA sample was amplified by real-time qPCR in triplicate.
