# Supplementary material for: Characterization of a novel theta-type replicon of indigenous plasmid pTE15 from Lactobacillus reuteri N16
Source: BMC Microbiol. 2022 Dec 12;22:298. doi: 10.1186/s12866-022-02718-4 (PMC9743546; doi:10.1186/s12866-022-02718-4)
Supplement: Supplementary file 2 — Additional file 2: Figure S6. (A) of original blots were from the red box with dash-line in lines 3–4 and lines 7–8 of panel C. (B) of original blots were from the blue box with dash-line in lines 1–2 and lines 3–4 of panel D. In panel D, lines 5–8 were from RCR-type plasmid pC194 of Staphylococcus aureus, which was not included in this manuscript. [file 12866_2022_2718_MOESM2_ESM.docx]

(C) (D)

1 2 3 4 5 6 7 8

1 2 3 4 5 6 7 8

rif.

S1

rif.

S1

− − + − − − + −

− + − + − + − +

Figure 6.

|  | 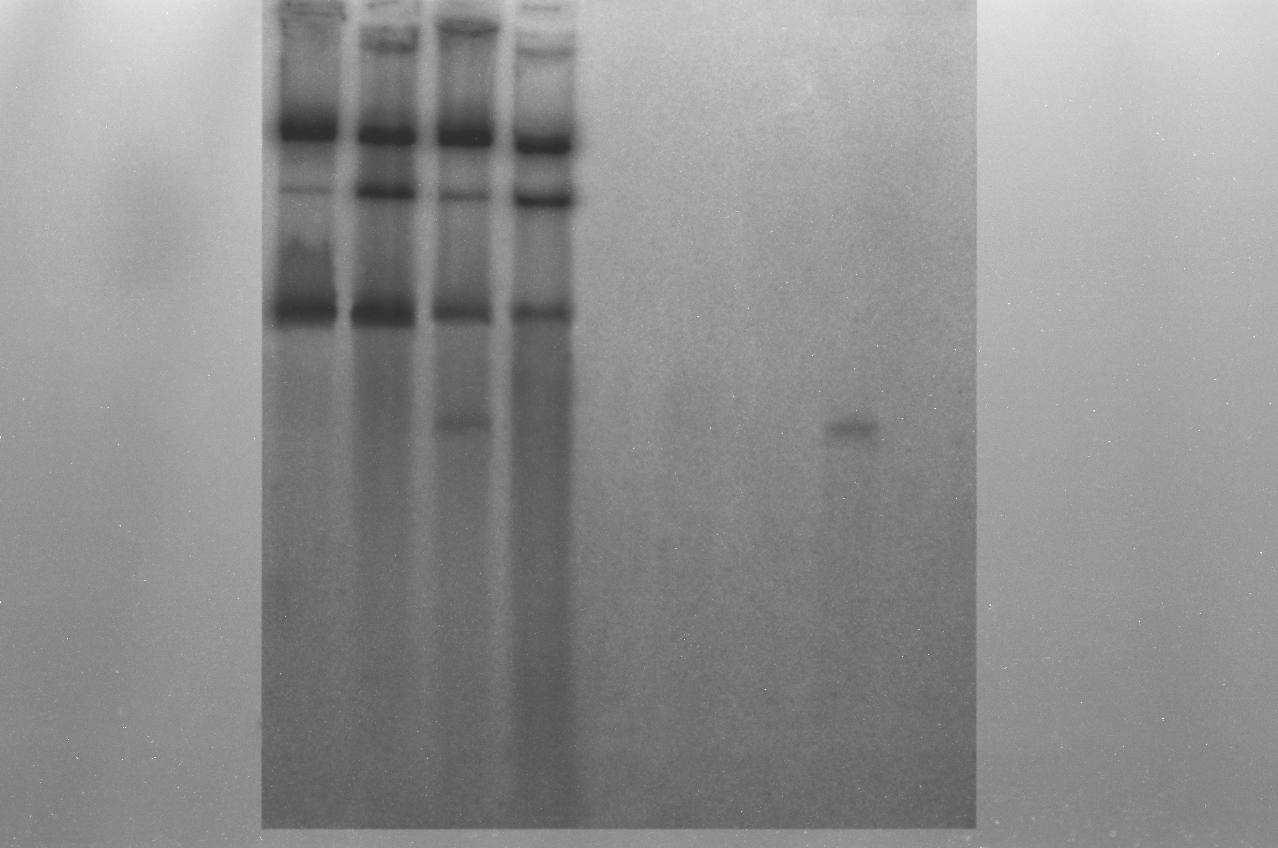 | 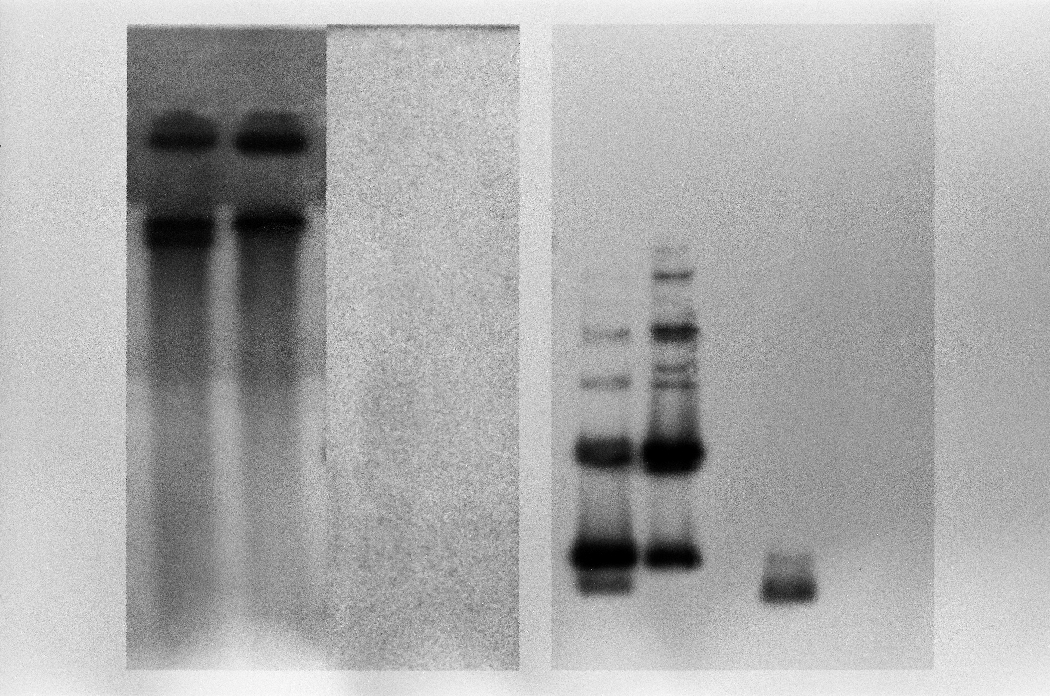  + − + − + − + −  − + − + − + − + |
| --- | --- | --- |

Explanation: Figure 6 (A) of original blots were from the red box with dash-line in lines 3-4 and lines 7-8 of panel C. Figure 6 (B) of original blots were from the blue box with dash-line in lines 1-2 and lines 3-4 of panel D. In panel D, lines 5-8 were from RCR-type plasmid pC194 of *Staphylococcus aureus*, which was not included in this manuscript.
